# Neuroprotective effect of triptolide on neuronal inflammation in rats with mild brain injury

**DOI:** 10.1016/j.ibneur.2024.05.007

**Published:** 2024-05-23

**Authors:** Zhanglu Fang, Guanghong Shen, Chengjian Lou, Benson O.A. Botchway, Qinglin Lu, Qining Yang, Nashwa Amin

**Affiliations:** a Department of Orthopaedics, Jinhua Municipal Central Hospital, Affiliated Jinhua Hospital, Zhejiang University School of Medicine, Jinhua, China; b Jinhua Maternal and Child Health Hospital, Zhejiang University School of Medicine, Jinhua, China; c Department of Neurosurgery, Fourth Affiliated Hospital, Zhejiang University School of Medicine, Yiwu 322022, China; d Institute of Systemic Medicine, Zhejiang University School of Medicine, Hangzhou 310058, China; e The Affiliated People’s Hospital of Hangzhou Medical College, Hangzhou Medical College, Hangzhou, China; f Department of Zoology, Faculty of Science, Aswan University, Egypt

**Keywords:** Mild traumatic brain injury, Neuro-inflammation, Autophagy, Triptolide

## Abstract

Concussions sustained while playing sports are a prominent cause of mild traumatic brain injury (mTBI), which is prevalent among teenagers. The early and intermediate stages of mild traumatic brain injury (mTBI) can be characterized by inflammation, neurodegeneration, and brain tissue edema, which can lead to permanent brain damage.

The present study investigated the therapeutic effects of triptolide in mTBI and brain damage recovery. After building mTBI model in male rat, triptolide administrated daily for 1 week in the treated group. On day 3 and day 7 of administration, hippocampus tissues were collected to evaluate inflammation and autophagy in the brain. The expressions of inflammatory factors interleukin (IL)-1β and tumor necrosis factor-alpha in serum were downregulated, while IL-10 expression was upregulated when compared with the mTBI group on day 3 and day 7. The expression of IL-10 on day 7 was higher than on day 3. Quantitative polymerase chain reaction (qPCR) analysis of inflammatory-related factors (i.e., *Il-1β* and nuclear factor-κB (*Nf-κb*), and western blot as well as immunofluorescence staining of autophagy-related proteins (i.e., LC3B) and aquaporin (AQP 4) showed lower expression on day 3 and day 7 in the triptolide-treated group. Moreover, NeuN immunostaining, and hematoxylin and eosin (HE) staining for hippocampus region revealed that the triptolide-treated group showed a decrease in damaged cells. Our findings emphasize the effectiveness of triptolide therapy after mild traumatic brain injury via modulating autophagy, attenuating inflammation and reduces edema by decreasing AQP 4 expression.

## Highlights

• TNF-α and IL-1β increased in the mTBI group in the hippocampus and serum.
• Triptolide attenuates neuroinflammation and neuronal damage in mTBI.
• LC3B as an autophagy marker increased in the hippocampus of the mTBI group.
• AQP4 as oedema increased obviously in the hippocampus of mTBI.
• Triptolide enhances IL-10 expression following mTBI in rats.

## Graphical Abstract

**Figure:**
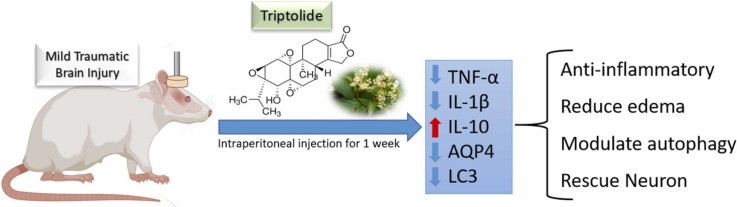

## 1 Introduction

Mild brain injury refers to minimal damage to the brain after 30 minutes of coma and is characterized by dizziness, headache, nausea and vomiting, and retrograde amnesia (Lefevre-Dognin et al., 2021) without significant signs of neurological examination (Wang et al., 2017). It frequently occurs in childhood, especially in adolescence. The incidence of mild brain injury has been on the rise in recent years mainly in high-income countries (HICs), especially in people above 65 years old (Capizzi et al., 2020). Numerous studies from the United States and New Zealand estimate that there are 500–800 new cases of TBI per 100,000 persons each year. Data from low- and middle-income countries (LMICs) are sadly severely lacking. According to a significant survey-based study that included participants from 8 LMICs, the prevalence of TBI was estimated to be less than 1 % in China and up to 15 % in Mexico and Venezuela. The majority of these findings are close to those from high-income nations (HINs). Therefore, ongoing attempts to gather as much trustworthy epidemiological data as possible on the incidence of mortality and disability from TBI in settings with limited resources are required (Dewan et al., 2018 Apr 27). Besides, the prevalence of incidence pediatric traumatic brain injury (pTBI) has been rising, with estimates of the annual frequency of hospitalizations for pTBI ranging from 50 to 300 per 100,000 kids. Children under the age of two and teens experience the highest incidences. In Europe, the incidence estimate for pTBI in 2014 was 349 per 100,000 kids ages 0–19 (Mitra et al., 2006). The primary cause of the damage is neuronal apoptosis and degeneration (Martin Cente). If treatment is delayed, this injury may impair adolescents' cognitive performance and balance, even if brain damage and clinical symptoms are less evident. The migration of peripheral leukocytes into the cerebral parenchyma and the activation of innate immune cells characterize the severe inflammatory response caused by traumatic brain injury (TBI). Neuronal survival and death are directly impacted by the invasion of neutrophils, monocytes, and lymphocytes at the site of damage (Postolache et al., 2020). Furthermore, upon arrival at the site of damage, activated microglia emit a variety of harmful chemicals such as proteases, nitric oxide, reactive oxygen species, chemotactic cytokines, and cytokines, which may exacerbate neuronal death (Hernandez-Ontiveros et al., 2013 Mar 26). Many mediators that are released after traumatic brain injury (TBI) exacerbate vasogenic and/or cytotoxic brain edema. These consist of free oxygen radicals, histamine, kinins, glutamate, lactate, H+, K+, Ca2+, nitric oxide, and arachidonic acid and its metabolites. The general cerebral edema that results from TBI is characterized by a combination of cytotoxic and vasogenic edema processes (McBride et al., 2014). Changes in the extracellular pH and ion concentrations, particularly potassium, sodium, and chloride, cause glial cells to expand following traumatic brain injury (TBI).3 The vasogenic edema brought on by the direct damage to the BBB mixes with the ensuing cytotoxic edema. Further ion shifts are brought on by cerebral ischemia, which is a reduction in blood supply to the affected brain region and exacerbates cytotoxic edema. Up until the brain swells excessively, causing irreversible brain damage or even death, a vicious cycle involving elements of both forms of edema may continue (McBride et al., 2014).

Abilities relating to coordination affect the normal growth and development of young people, as well as their social life (Fried et al., 2022). Furthermore, after the initial episode of mTBI, the susceptibility to a second traumatic injury increases (Fried et al., 2022, Laurer 1 et al., 2001), thus, aggravating the traumatic effects. Presently, clinical treatments of mild brain injury involve several hormonal shocks and drugs to counter some of the detrimental repercussions, such as edema (Capizzi et al., 2020). Herbal medicines have been reported to have immune promotion, anti-inflammatory, and neuroprotective effects on the central nervous system, however, these medicines have some side effects including immunodeficiency, hypertension, hyperglycemia, bleeding, anemia, and life-threatening allergic reactions (Pilipović et al., 2021). For example, Triptolide is the main active substance of Tripterygium wilfordii, having anti-inflammatory, immunosuppressive, anti-tumor, and anti-fertility effects (Shamon et al., 1997). It is used in treating rheumatoid arthritis, nephritis, asthma, systemic lupus erythematosus, skin diseases, and several autoimmune and inflammatory diseases (Hikim et al., 2000). Due to its water insolubility and significant toxicity to the gastrointestinal, renal, cardiac, hepatic, hematopoietic, and reproductive systems, triptolide was not commonly employed in clinical settings (Wong et al., 2008). Triptolide has been linked to reports of male rat infertility (Lee et al., 2020). The clinical efficacy of triptolide is constrained by its weak water solubility. For instance, triptolide injections intravenously call for the preparation of the drug with a solubilizing agent such as Cremophor EL. Triptolide, has been linked to significant clinical side effects in certain patients, including severe anaphylactoid hypersensitivity reactions, hyperlipidemia, aberrant lipoprotein patterns, erythrocyte aggregation, and peripheral neuropathy (Wei et al., 2019). However, at nanomolar concentration, it inhibits the formation of inflammatory cytokines (Fang et al., 2021) as well as the expression of damage-associated proteases and transcriptional factors (Pan and Xu, 2020). Triptolide has been demonstrated to suppress IL-12 and IL-23 expression in dendritic cells (DCs) and a human monocytic cell line (THP-1 cells), and to lessen IFN-gamma-induced CD80 and CD86 expression (Fehily and Fitzgerald, 2017). Triptolide's ability to modulate the immune system was recently demonstrated to decrease the expression of Il-12 and IL-23 in antigen-presenting cells (APCs) via CCAAT/enhancer-binding protein alpha (Lozano et al., 2015). Triptolide reduced TBI-induced increases in contusion volume, cell apoptosis, edema, and levels of pro-inflammatory mediators in the brain. Triptolide reduced the TBI-induced reduction in brain levels of the anti-inflammatory cytokine interleukin-10. Importantly, triptolide enhanced neurobehavioral outcomes related to motor, sensory, reflex, and balance function (Lee et al., 2012). Over the last three decades, over a thousand individuals with various autoimmune and inflammatory disorders have been treated with TWHF extracts in clinical studies and treatment in China. In the majority of these investigations, the T2 extract, which known as multiglycoside or polyglucoside, was used in conjunction with other treatments. Triptolide has been shown to reduce inflammation and improve renal function in patients with diabetic nephropathy (Ziaei and Halaby, 2016). Triptolide has been shown to be an effective type of treatment for asthma by reduction of IL-5 production. More clinical trials with bigger sample sizes are needed to investigate the efficacy of triptolide and its analogues as treatments for inflammatory and autoimmune disorders (Ziaei and Halaby, 2016). Although Tripterygium wilfordii has a wide range of bio-activities and pharmacological effects both in vivo and in vitro, it has been limited in clinical use due to frequent reports of multi-target toxicity.

Commercial preparations of T. wilfordii have caused 633 adverse responses, including 53 severe cases of reproductive toxicity, hepatotoxicity, and renal cytotoxicity (Li et al., 2014). Furthermore, 271 patients with rheumatoid arthritis experienced side effects from T. wilfordii, including digestive tract complaints and irregular menstruation. However, further research is needed to understand the processes that regulate triptolide's hazardous effect. More strict randomized double-blind clinical trials are especially necessary. Previous research has shown that the hippocampus is extremely sensitive to brain injury, both in animal models of TBI and in human patients. Although the hippocampus is rarely directly mechanically harmed by a head injury, it does experience atrophy and deficiencies in long-term potentiation (LTP), which is a prolonged increase in synaptic strength that is thought to be a model of learning and memory (Osier and Dixon, 2016, Biegon, 2021). In this study, an experimental animal model of mild brain injury is treated with triptolide, and its effects on hippocampal neuronal autotroph-related proteins and inflammatory factors are observed.Our findings provide a possible new interventional agent for mild traumatic brain injury by using triptolide.

## 2 Materials and methods

### 2.1 Animals

Adult male Sprague-Dawley rats were used in all the experiments (10 weeks old, 300–350 g), and provided by the Animal Center of Zhejiang Academy of Medical Science. Before the experiment, animals were reared in the experimental animal facility for a week. All experiments were conducted following the National Institutes of Health Guide for the Care and Use of Laboratory Animals. The Ethics Committee for Animal Research at Zhejiang University approved the experimental procedures.

### 2.2 The mTBI model and triptolide administration

Rats were divided randomly into 6 groups, 29 rats in each group: 3 days; sham (normal rats without model or triptolide treatment), mTBI (model), and model with triptolide treatment (mTBI + TP). 7 days; sham (normal rats without model or triptolide treatment), mTBI (model), and model with triptolide treatment (mTBI + TP). The sham group rats were left untreated. The rats in the triptolide treatment group were intraperitoneally injected with 0.2 mg/kg triptolide (Osier and Dixon, 2016) for a week.TP (Med Chem Express, USA#39,836) was dissolved in DMSO at a concentration of 2 mg/mL and diluted with normal saline to a final concentration of 0.2 mg/ mL. The rats in mTBI and sham groups were intraperitoneally injected with 0.2 mg/kg of dimethyl sulfoxide (DMSO) 0.1 %. All actions were performed once daily and continued for a week. The animals room temperature was set between 24 ± 1 ℃, humidity at 55 %, and a 12 h light-dark cycle. Standard food pellets and tap water were made available during the experiments. Referring to the controlled cortical impact injury (CCI) method developed by *Osier* et al. an mTBI model was created using RWD's brain injury impactor (Lee et al., 2012). The brief steps for model production are as follows: Rats were anesthetized by intraperitoneal injection of 1 % pentobarbital sodium at a dose of 35 mg/kg, placed in a prone position on a stereotactic headrest, incised in the midline under sterile conditions scalp, exposing the right parietal bone. Open 2.5 mm next to the midline of the sagittal suture, with −2 mm behind the anterior fontanel as the impact center. The impact head of the craniocerebral injury impactor was placed at a 15 ° angle to the sagittal plane of the rat head. By setting the impactor, a 4 mm diameter impactor was used to produce mTBI at a collision speed of 3 m/s, a depth of 1 mm, and a collision time of 180 ms. The sham group rat underwent the above surgery and anesthesia procedures, but there was no impact injury process.

### 2.3 Hematoxylin and eosin staining (H&E)

HE staining was used to evaluate morphological alterations in hippocampus following mTBI. Following the perfusion by paraformaldehyde (PFA) 4 % and freezing of rats’ brains, coronal 10-μm sections were taken. The fixed sections were placed in distilled water for 2 minutesAfter deparaffinization and rehydration, tissue sections were stained with hematoxylin solution for 8 minutes, followed by acidic ethanol (1 % HCL/70 % ethanol) for a few seconds, rinsed with running water to remove excess stain, and then stained with eosin solution for 5 minutes. After, the sections were stained with eosin solution for 5 min followed by dehydration with a graded alcohol series before being cleared in xylene. The slides were sealed with neutral balsam and inspected with a microscope. Changes in the hippocampus were observed under a light microscope. 3 different samples each group were used. Survival neuron cells counted by image pro plus.

### 2.4 Western blotting

Total proteins of the hippocampus tissue were extracted with 1 % PMSF (Phenylmethanesulfonyl fluoride, Beyotime ST505) in 1-mL ice-cold RIPA buffer with protease inhibitor cocktail EDTA-free and phosphatase inhibitors. After homogenizing and centrifuging at 12,000 rpm for 20 min at 4 °C, supernatant proteins were stored at −80 °C. Protein concentration was measured by BCA kits (KeyGEN, Nanjing, China), the protein supernatant was collected, and 5 × SDS-PAGE protein upload buffer was added to configure a mixture with the protein supernatant. An equal amount of protein mix was added to a 10 % gel for electrophoresis and then transferred to a PVDF membrane. The PVDF membrane was incubated with the designated primary antibody buffer at 4 ◦C overnight with slow shaking after being submerged in TBST (sealing solution) containing 5 % skimmed milk powder for 3 h. The initial antibodies were GAPDH (1:10000, Baoke Bio, China), LC3 (1:400, CST, China), and AQP4 (1:500, CST, China). In the following thirty minutes, the membranes were cleaned three times with TBST.

Then, according to the host of the primary antibody, the appropriate secondary antibody was chosen: an anti-goat anti-rabbit (Baoke, China, 1:5000). It was then incubated for two hours at room temperature and then washed. Finally, all membranes were sequentially and uniformly dosed with the appropriate amount of developer and placed into the chemiluminescence imager with the parameters set for the assay, which were normalized to β-actin loading control (Bio-Rad).

### 2.5 Enzyme-linked immunosorbent assay

Rats were intraperitoneally injected with 0.2 mg/kg triptolide for 7 days. On days 3 and 7, eight rats in each group were anesthetized with 2 % pentobarbital (0.3 mL/100 g). Blood was taken from the eyelids. The serum was separated by centrifugation and subjected to enzyme-linked immunosorbent assay (ELISA) by specific ELISA kits (all from Aimeng Youning, China). The expression levels of interleukin 10 (IL-10), tumor necrosis factor-alpha (TNF-α), and IL-1β in rat serum were detected by ELISA. The prepared samples and standards were added, and after adding the enzyme-labeled reagent to each well, the cells were incubated (37 °C, 60 minutes) and washed 5 times with a diluted washing solution. The coloring solutions (i.e., A and B) were added, the color was developed at 37 °C for 15 minutes, and the stop solution was added. Afterward, the optical density (OD) was read within 15 minutes.

### 2.6 Immunofluorescence staining

Rats were cardiac perfused with 50 mL 0.9 % normal saline to flush their vascular blood and then perfused with 4 % PFA. After perfusion, brain tissue was obtained and conserved in 4 % PFA for a day, and the fixation fluid was replaced with a 30 % sucrose solution. The embedding and frozen tissue sections were performed using a freezing microtome. After washing with PBS, the slices were incubated for 2 hours in the blocking solution. Then, the primary antibodies; LC3B (Cell Signaling Technology, 1:200), AQP4 (Abcam, 1:400) and NeuN (Cell Signaling Technology, 1:500) were added, and slices were incubated overnight at 4 °C. The next day, all slices were washed 3 times with PBS for 5 minutes each and the secondary antibody (1:500) was added in the dark, the sample was sealed at room temperature for 2.5 hours. The slices were observed under the Olympus VS120 (Olympus Corporation of Japan) fluorescence microscope. Three samples were randomly selected from each group the same target area was observed, and images were analyzed using image pro plus software. The average of the three fields of view was used to calculate the integrated optical density (IOD) and positive index (Wong et al., 2008), which was the ratio of IOD to image resolution.

### 2.7 Real-time quantitative PCR

The fresh brain tissue was taken and the hippocampal tissue was separated and immediately placed at –80 °C for quantitative PCR (qPCR). Approximately 30 mg of tissue was taken from the hippocampal tissue of each group of mice using RNase-free surgical instruments in an RNase-free environment. Total RNA from the tissues was extracted. RNA extraction and quantitative real-time PCR (RT-qPCR) assay: Total mRNA was extracted from brain tissue using TRIzol® reagent (Invitrogen; Thermo Fisher Scientific, Inc.). RNA quality was determined using a NanoDrop 2000c spectrophotometer (Thermo Fisher Scientific, Inc.). Total RNA (3 µg) was reverse transcribed into cDNA using the Bestar qPCR RT kit (DBI Bioscience). The temperature protocol used for reverse transcription was 37 °C for 15 min and 98 °C for 5 min. Subsequently, qPCR was performed using the Bestar qPCR MasterMix (DBI Bioscience) and an ABI 7500 system (Applied Biosystems; Thermo Fisher Scientific, Inc.), according to the manufacturer's protocol. The following thermocycling conditions were used for qPCR: 95 °C for 2 min; 95 °C for 10 sec, 60 °C for 34 sec, 72 °C for 30 sec; and the solubility curve was obtained at 98 °C, a total of 40 cycles; dissolution curve at 95 °C for 1 minute, 55 °C for 1 minute, and 55 °C at – 98°C (10 s/cycle, 0.5 °C/cycle) for 86 cycles. The data were collected using the Bio-Rad CFX96 touch fluorescence quantitative PCR instrument, and each sample was divided into three duplicate wells. The Ct average of the samples was measured and calculated using computer software. The relative mRNA expression was calculated using the formula 2^–ΔΔCt^ (ΔΔCt= (Ct value of the target gene of the experimental group – Ct value of the reference gene of the experimental group) – (Ct value of the target gene of the sham group – Ct value of the reference gene of the sham group). The primers used for qPCR are presented in Table 1.

**Table 1:** Primer Sequence.

**Gene**	**Forward primer sequence 5’-3’**	**Reverse primer sequence 5’-3’**
***Il-1β***	CAGGAAGGCAGTGTCACTCA	AGACAGCACGAGGCATTTTT
***Nf-κb***	CCCCCTGAGAAAGAAACACA	ACACTGTCCCCGTTCTCATC
***Gapdh***	AGTGCCAGCCTCGTCTCATA	ACGACATACTCAGCACCAGC

### 2.8 Statistical analysis

Data were statistically analyzed using the SPSS 20.0 statistical software. Data are expressed as SEM. One-way analysis of variance was used for comparison among groups. The difference was evaluated by one-way ANOVA with Tukey’s tests between drug treatment and control group in mTBI models. Regarding the data of multiple groups, the difference was analyzed using the two-way ANOVA with Bonferroni’s post-test. A *P* < 0.05 was considered statistically significant.

## 3 Results

### 3.1 Pathological changes in the hippocampus of mTBI rats

**Fig. 1:**
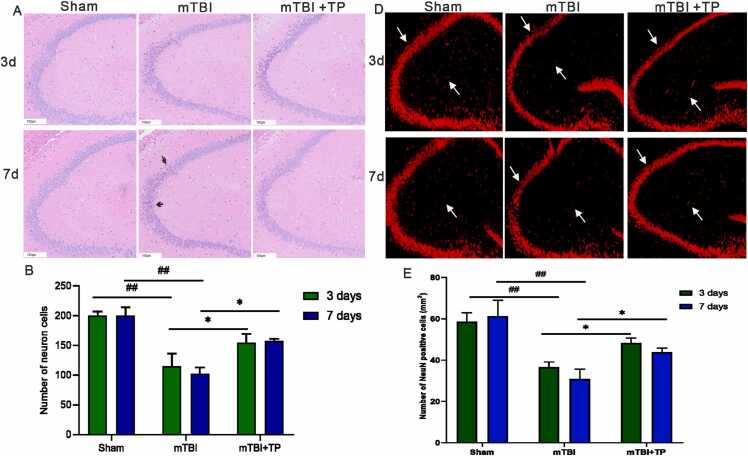
Pathological changes in the hippocampus of mTBI rat. A.HE staining showed that the swelling and rupture of rat cells were decreased after triptolide treatment. B. number of neuron cells. Swelling and ruptured nerve cells can be seen in the image, as shown by the black arrow. ^##^*P* < 0.01 mTBI compared with the sham group in both day 3 and day 7; *P < 0.05, mTBI+TP compared with the mTBI group day 3 and day 7. scale bar 100 µm, n=3. C. NeuN immunostaning (red). D. NeuN positive cells.comparison between groups on day 7, and day 3 each group. ^##^*P* < 0.01 mTBI compared with the sham group in both day 3 and day 7; *P < 0.05, mTBI+TP compared with the mTBI group day 3 and day 7. Data presented as mean + SD. n=5. The *,and ^#^, indicate statistically significant differences.

The HE staining results showed that the hippocampus of the sham group had clear edges, uniform cell arrangement, pink extracellular matrix, and elliptical nuclei. In the mTBI group, the number of neurons was reduced, cell morphology was round, and nuclear fragmentation was observed. Moreover, the number of damaged cells was increased on day 7 when compared to day 3, accompanied by a blurring of cell edges. In the mTBI + TP group, the cell margin and nuclear fragmentation were observed in the early stage (day 3), but the number of normal neurons was higher than in the mTBI group during the same period. In the advanced stage (day 7), the swelling of cells was reduced, and normal was observed (Fig. 1 A-B). These results confirmed by NeuN staining (Fig. 1 C-D), which demonstrated a lower number from Neun positive cells compared with sham groups after both 3 days and 7 days. However, TP treatment increased the positive cells compared with mTBI model.

### 3.2 The effect of triptolide on inflammatory factors after mTBI

**Fig. 2:**
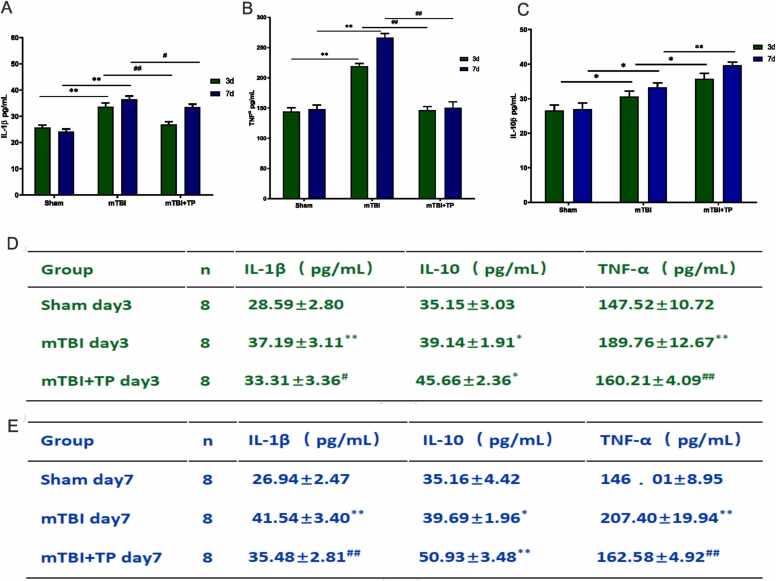
Inflammatory-related factors A. IL-1β, B. TNF-α and C. IL-10; changes after triptolide treatment. D&E individual raw data of each factors after 3 days and 7 days. Comparison between groups on day 3, comparison between groups on day 7, and day 3 and day 7 for each group. Data presented as mean + SD. The *, ^#^, indicate statistically significant differences. IL-1β; mTBI Vs. Sham day3 ^**^P < 0.01, day 7 ^**^P < 0.01^,^ mTBI+TP Vs. mTBI; day3 ^##^*P* < 0.01, day 7 ^#^*P*^<^ 0.05, TNF-ɑ; mTBI Vs. Sham day3 ^**^P < 0.01, day 7 ^**^P < 0.01, mTBI+TP Vs. mTBI; day3 ^##^*P* < 0.01, day 7 ^##^*P*^<^ 0.01, IL-10; mTBI Vs. Sham day3 *P < 0.05^,^ day 7 *P < 0.05, mTBI+TP Vs^.^ mTBI; day3 ^**^P < 0.01, day 7 ^**^P < 0.01, n=10.

In assessing whether triptolide could reduce inflammatory factors, we analyzed IL-1β and TNF-α expression (Fig. 2) in the serum. The expression of these two inflammatory factors was significantly increased in the mTBI and mTBI + TP groups when compared with the sham group at both time points (i.e., days 3 and 7) (*P* < 0.05). Furthermore, the expression of these two inflammatory factors was considerably lower in the mTBI + TP group than in the mTBI group (*P* < 0.05). No significant difference was found between day 3 and day 7 regarding these two groups. Moreover, compared with the mTBI + TP group, the expression of inflammatory factors continued to increase in the mTBI group on day 7, with the difference being statistically significant (*P* < 0.01). The IL-10 expression in the serum was significantly higher in the mTBI + TP group than in both mTBI and sham groups (*P* < 0.01). Triptolide also promoted the expression of IL-10 (Fig. 2).

### 3.3 Decreased expression of inflammatory-related genes IL-1β and NF-κB at the mRNA level

**Fig. 3:**
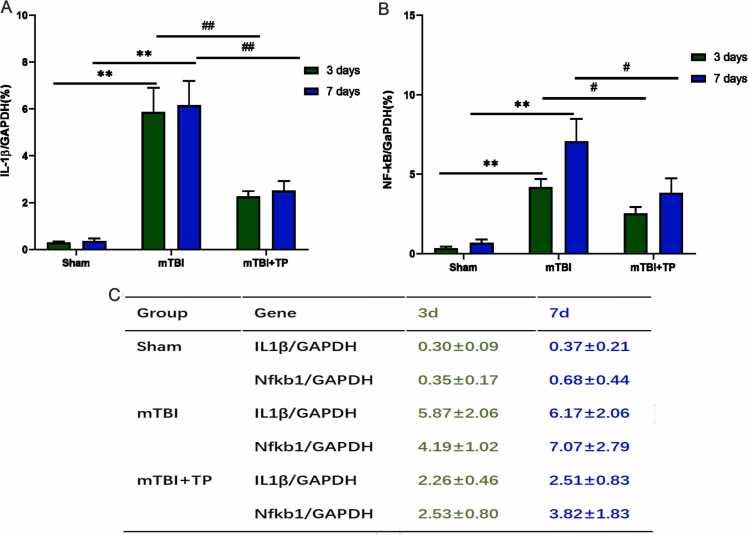
Triptolide downregulated IL-1β and NF-κB expressions. The relative mRNA expression was calculated using the formula 2–^*ΔΔ*^Ct. Comparison between groups on day 3, comparison between groups on day 7, and day 3 and day 7 for each group. Data presented as mean + SD. The indicate statistically significant differences. IL-1β; mTBI Vs. Sham day3 ^**^P < 0.01, day 7 ^**^P < 0.01^,^ mTBI+TP Vs. mTBI; day3 ^##^*P* < 0.01, day 7 ^#^*P*^<^ 0.05, NF-kB; mTBI Vs. Sham day3 ^**^P < 0.01, day 7 ^**^P < 0.01, mTBI+TP Vs. mTBI; day3 ^##^*P* < 0.01, day 7 ^##^*P*^<^ 0.01, n=8.

The expression of IL-1β and NF-κB mRNA was significantly higher in the mTBI and mTBI +TP groups than in the sham group (*P* < 0.05). Moreover, inflammatory-related gene expressions were significantly lower in the mTBI + TP group than in the mTBI group (*P* < 0.05). The three groups were compared before and after the interval, showing no significant difference. These findings suggest that triptolide may inhibit the expression of inflammatory-related genes (i.e., IL-1β and NF-κB) at the genetic level (Fig. 3).

### 3.4 Triptolide downregulates the expression levels of LC3B and AQP4 in the hippocampus

**Fig. 4:**
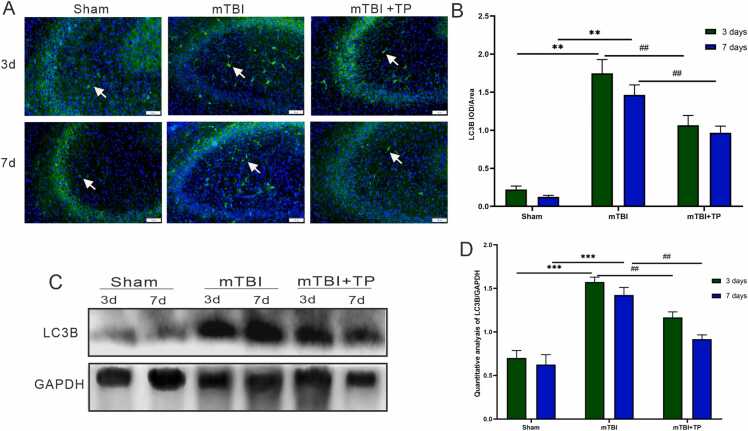
LC3B expression changes after triptolide treatment at different time points. (A): Representative LC3B positive (green) and DAPI (blue) merged image is shown. In the mTBI group, the positive expression index of autophagy protein LC3B was higher than both sham and mTBI + TP groups. The autophagy protein experimental group can be seen in the image. Scale bar = 50 μm. Immunofluorescence showed decreased expression of autophagy and aquaporin in rats treated with triptolide. (B): Comparison between groups on day 3, comparison between groups on day 7, and day 3 and day 7 for each group. Data presented as mean + SD. The *, #, indicate statistically significant differences. mTBI Vs. Sham day3 ^**^P < 0.01, day 7 ^**^P < 0.01^,^ mTBI+TP Vs. mTBI; day3 ^##^*P* < 0.01, day 7 ^##^*P*^<^ 0.01 day 7, n=8. C. LC3B Western blot, D. Quantitative analysis of LC3B; mTBI Vs. Sham day3 ^**^P < 0.01, day 7 ^**^P < 0.01, mTBI+TP Vs. mTBI; day3 ^##^*P* < 0.01, day 7 ^##^*P*^<^ 0.01 day 7, n=3.

**Fig. 5:**
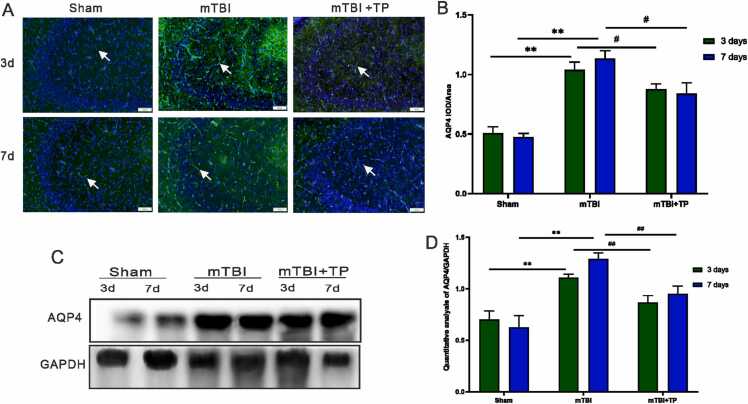
AQP4 expression changes after triptolide treatment at different time points. (A): Representative AQP4 positive (green) and DAPI (blue) merged image is shown. In the mTBI group, the positive expression index of AQP4 was higher than the sham and mTBI + TP groups. The Aquaporin of an experimental group can be seen in the image. Scale bar = 50 μm. Immunofluorescence showed decreased expression of autophagy and aquaporin in rats treated with triptolide. (B): Comparison between groups on day 3, comparison between groups on day 7, and day 3 and day 7 for each group. Data presented as mean + SD, n=8. The *, #, indicate statistically significant differences. mTBI Vs. Sham day3 ^**^P < 0.01, day 7 ^**^P < 0.01^,^ mTBI+TP Vs. mTBI; day3 ^#^*P* < 0.05, day 7 ^#^*P* < 0.05 day 7, n=8. C. LC3B Western blot, D. Quantitative analysis of LC3B; mTBI Vs. Sham day3 ^**^P < 0.01, day 7 ^**^P < 0.01, mTBI+TP Vs. mTBI; day3 ^##^*P* < 0.01, day 7 ^##^*P*^<^ 0.01 day 7, n=3.

In the mTBI group, the positive expressions of autophagy protein (LC3B) and AQP4 protein which represents an indicator for edema, were higher than the sham and mTBI + TP groups, with a statistically significant difference (*P* < 0.05). The number of LC3B and AQP4 positive indices did not change significantly in the sham group. The positive indices of both LC3B and AQP4 were higher in the mTBI + TP group than in the sham group and were significantly reduced when compared with the mTBI group (*P* < 0.01). Both LC3B and AQP4 positive indices did not continue to increase (Fig. 4 A-B and Fig. 5 A-B). The above findings agreed well with immunoblotting results of both (LC3B) and AQP4 protein (Fig. 4 C-D and Fig. 5 C-D).

## 4 Discussion

Traumatic brain injury is a leading cause of mortality and disability, particularly among young people, and a serious public health concern around the world. Epidemiological studies consistently show that men have a higher incidence of TBI than women, with the likelihood of sustaining one being 2.22 times higher (Biegon, 2021). The reported frequency of TBI in the general population is 16.7 % for males and 8.5 % for females. Males make up around 59 % of all reported TBI-related medical visits in the United States. Our research included the development of a mTBI male rat model as well as the use of triptolide to investigate its effects on hippocampal neuronal healing. In this study, the expression of inflammatory factors IL-1β and TNF-α were markedly lower after treatment with triptolide in mild brain injury (Fig. 2). However, the effect was significant after 1 week, indicating that triptolide could alleviate the inflammatory response. This protective effect might be related to its ability to act by inhibiting the NF-κB signaling pathway (Yang et al., 2022). NF-κB is a transcription factor that controls the expression of target genes involved in cell proliferation and inflammation. The activation of NF-κB following brain injury is accompanied by TNF-α and IL-1β expressions (Lanis et al., 2017). Therefore, triptolide may inhibit the expression of inflammatory factors by inhibiting NF-κB. Moreover, triptolide promotes the expression of the inflammatory protective factor, IL-10, thus allowing further control of inflammation and avoiding exacerbation of damage (Sun, 2017). Moreover, in the present study, the expression of autophagy-related proteins was decreased following treatment with triptolide. After 1 week, the expression was still low, which might be related to the inhibitory effect of triptolide on macrophages, resulting in a decrease in LC3B expression. Furthermore, following the employment of triptolide in treating mild brain injury in rats, the AQP4 expression in brain tissue after both 3 and 7 days was significantly reduced (Fig. 6), suggesting that triptolide is effective in attenuating edema following mTBI model. Nevertheless, triptolide affected brain tissue edema and could effectively mitigate secondary damage after brain injury. Moreover, the mRNA expression levels of NF-κB and IL1-β in the hippocampus of rats were considerably lower after triptolide treatment (Fig. 4).

Traumatic brain injury (TBI) is a generic term for an intracranial injury caused by an external mechanical force that destroys the brain structure or a change in its functioning (Hou et al., 2017). TBI has a variety of causes and outcomes. TBI is caused by a variety of mechanisms, including blunt forces (ranging from bumps to blows), inertial loads, penetrating wounds, and blast injuries (Silver et al., 2019). The level of injury varies, just as the causes of TBI differ. Immediate injury following a TBI can cause problems ranging from an unrecognized bruise with transient impairments to hemorrhage, or brain malfunction resulting in permanent disability or even death (Mira et al., 2021). Throughout assessing prospective TBI patients, it is critical to obtain an accurate history of injury and complications during injury (including loss of consciousness), to evaluate and manage symptoms (Osier and Dixon, 2016, Kim et al., 2015), and to do serial examinations throughout the post-injury period (Mira et al., 2021). Significant volume loss has been observed in clinical TBI patients, and the hippocampus is particularly susceptible to TBI (Shokouhi et al., 2020, Lisman et al., 2017). The hippocampus also plays a role in cognition, memory, and navigation (Wang et al., 2021a). Our research focused on hippocampal alterations because of the important pathophysiological mechanisms connected to the hippocampus in traumatic brain injury. Neuro-inflammation, a pathological reaction of the brain to different stimuli (such as injury, microbial infection, and chemical actions), is the main phenomenon in neuronal destruction. The expression of inflammatory markers (i.e., IL-1β and TNF-α) was much higher in studies on neuronal injury, cerebral ischemia, and inflammation, which intensified the inflammatory response (Lisman et al., 2017, Wang et al., 2021a, Carbonell et al., 1998) and promoted the transcription of IL-1β and NF-κB in brain tissue (Carbonell and Grady, 1999). After a traumatic brain injury (TBI), the central and peripheral nerve systems respond to inflammation in a complicated and dynamic way that is impacted by a person's age, sex, the location and severity of the damage, subsequent injury cascades, and heredity. The post-injury inflammatory response is becoming implicated as a major mediator in the long-term healing from traumatic brain injury (Smith et al., 1997). Experimental TBI disrupts numerous biological pathways, leading to progressive neurodegeneration including as atrophy, neuronal loss, and axonal degeneration. This neurodegeneration is frequently linked to neuroinflammation, which includes macrophage reactivity (Smith et al., 1997).

Moreover, ROS induced the expression and activation of the autophagy protein, LC3B, and stimulated the formation of autophagosomes, resulting in cell death associated with caspase-3 activation (Sun and Yue, 2019, Ahsan et al., 2021). IL-10 is an anti-inflammatory cytokine that inhibits inflammation-induced metabolic processes in macrophages and eliminates mitochondrial autophagy by stimulating damaged macrophages and removing dysfunctional mitochondria (Eddie Ip, 2019). Thus, it (i.e., IL-10) can cause the dysregulation of the NLRP3 inflammasome production and reduce IL-1β formation, hence inhibiting the progression of an inflammatory response (Li et al., 2021). Consequently, inhibiting neuro-inflammation can be a novel strategy for treating neuronal damage.

Increased intracranial pressure caused by cerebral edema could aggravate brain injury. Intracranial edema is divided into two types: cytotoxic edema (intracellular edema) and vasogenic edema (extracellular edema) (Koenig, 2018). Following TBI, an immune gene expression program is activated, which influences the course of the ensuing inflammatory cascade (Ehsan Dadgostar). Antigen presentation [CD74, CD86, major histocompatibility complex (MHC) II], phagocytosis [C3, C4, Fc gamma receptor (FcgR) and FCGR4], astrocyte activation [Aquaporin (AQP)4 and GFAP], chemotaxis [chemokine ligand (CCL)2, CCL4, chemokine (C-X-C motif) ligand (CXCL), Cytokine signaling [IL-1, IFN-, IL-6, IL-12, IL-10, and TGF-] is interestingly elevated in both moderate and severe TBI (Zhang et al., 2019), suggesting that they are unlikely to be significant mediators of TBI outcome severity. Early after damage, toxic edema sets up, with astrocytes being the primary cells impacted. Vasogenic edema is the predominant condition in the late post-injury period as a result of the blood-brain barrier being destroyed (Lagraoui et al., 2012). Tissue edema and cell swelling are caused by AQP4 overexpression and activation (Zhang et al., 2016). The primary isoform of AQP4 expressed on astrocytes and ependymal cells has been linked to cerebral edema brought on by traumatic brain injury (Lagraoui et al., 2012). The direction of water transport can be regulated by the AQP4 in response to an osmotic gradient. Consequently, an excessive amount of water molecules may enter the cells if AQP4 is overexpressed, leading to edema and neuronal rupture (Zhang et al., 2016).

Moreover, this would further aggravate the damage to brain tissue. The AQP4 is a significant player in neuroinflammation (Szczygielski et al., 2021). Studies have shown that the AQP4 can promote astrocytes through the sphingosine kinase 1 (SPHK1) / mitogen-activated protein kinase (MAPK) / protein kinase B (AKT) pathway (Dai et al., 2018). Glial cells release proinflammatory factors, which further exacerbate inflammatory response after brain injury, suggesting that the inhibition of the AQP4 protein gene transcription may not only inhibit brain edema but also impair inflammation (Wu et al., 2019). Thus, controlling brain edema could be the key to treating brain damage.

Progesterone-type neurosteroid hormones have been extensively used in neurodegenerative diseases. They have significant anti-inflammatory and neuroprotective effects by promoting synaptic function and myelination (Kolatorova et al., 2022). Triptolide has a chemical structure that is highly similar to progesterone neurosteroids, implying that it has the same potential to cure brain injuries as progesterone neurosteroid hormone (Wang et al., 2021b). Triptolide can stimulate the hypothalamic-pituitary-adrenal axis and generate endogenous glucocorticoids, implying that it could be used to treat brain injury (Lu et al., 2016). Our findings suggest that triptolide could hinder neuro-inflammation and offer multi-faceted protection of neuronal survival in mild brain injury. In the future study, the behavioral improvement and a correlation of AQP4 with edema in the cortex and hippocampus of mTBI models could be confirmed after TP treatment. The *Cx3cr1*^-cre^:: Ai09 mice could be used to estimate the microglia activation after mTBI.

## 5 Conclusion

Triptolide was found to lessen inflammation in mTBI rat model in this study. Triptolide can also reduce the expression of autophagy-related proteins and reduce aquaporin opening to protect neurons. This protective effect has been found to continue for one week following triptolide treatment. However, due to the complexities of regulating neuro-inflammation as well as neuronal repair and regeneration, further research about the mechanism and effect of triptolide in treating mTBI injuries is required.

## Funding

This research was funded by Jinhua Science and technology research plan project, grant number (2022–3–095).

## CRediT authorship contribution statement

**Qining Yang:** Funding acquisition, Project administration. **Nashwa Amin:** Software, Writing – original draft, Writing – review & editing. **Zhanglu Fang:** Investigation. **Benson O.A. Botchway:** Conceptualization. **Qinglin Lu:** Visualization. **Guanghong Shen:** Formal analysis. **Chengjian Lou:** Validation.

## Declaration of Competing Interest

The authors declare that the research was conducted in the absence of any commercial or financial relationships that could be construed as a potential conflict of interest.
